# Spatial scale and structure of complex life cycle trematode parasite communities in streams

**DOI:** 10.1371/journal.pone.0241973

**Published:** 2020-11-24

**Authors:** Sally A. Zemmer, Jillian T. Detwiler, Eric R. Sokol, Jeronimo G. Da Silva Neto, Jennie Wyderko, Kevin Potts, Zachary J. Gajewski, Lea V. Sarment, E. F. Benfield, Lisa K. Belden

**Affiliations:** 1 Biological Sciences, Virginia Polytechnic and State Institute, Blacksburg, Virginia, United States of America; 2 Biological Sciences, University of Manitoba, Winnipeg, Manitoba, Canada; Irstea, FRANCE

## Abstract

By considering the role of site-level factors and dispersal, metacommunity concepts have advanced our understanding of the processes that structure ecological communities. In dendritic systems, like streams and rivers, these processes may be impacted by network connectivity and unidirectional current. Streams and rivers are central to the dispersal of many pathogens, including parasites with complex, multi-host life cycles. Patterns in parasite distribution and diversity are often driven by host dispersal. We conducted two studies at different spatial scales (within and across stream networks) to investigate the importance of local and regional processes that structure trematode (parasitic flatworms) communities in streams. First, we examined trematode communities in first-intermediate host snails (*Elimia proxima*) in a survey of Appalachian headwater streams within the Upper New River Basin to assess regional turnover in community structure. We analyzed trematode communities based on both morphotype (visual identification) and haplotype (molecular identification), as cryptic diversity in larval trematodes could mask important community-level variation. Second, we examined communities at multiple sites (headwaters and main stem) within a stream network to assess potential roles of network position and downstream drift. Across stream networks, we found a broad scale spatial pattern in morphotype- and haplotype-defined communities due to regional turnover in the dominant parasite type. This pattern was correlated with elevation, but not with any other environmental factors. Additionally, we found evidence of multiple species within morphotypes, and greater genetic diversity in parasites with hosts limited to in-stream dispersal. Within network parasite prevalence, for at least some parasite taxa, was related to several site-level factors (elevation, snail density and stream depth), and total prevalence decreased from headwaters to main stem. Variation in the distribution and diversity of parasites at the regional scale may reflect differences in the abilities of hosts to disperse across the landscape. Within a stream network, species-environment relationships may counter the effects of downstream dispersal on community structure.

## Introduction

Metacommunity paradigms have broadened the scope of community-level investigations by addressing the roles of both local (e.g. within-site factors) and regional scale processes (e.g. dispersal) in driving community structure [1, 2]. Most initial research on metacommunity dynamics focused on systems with discrete habitat patches, such as ponds or forest fragments, where overland dispersal can be clearly defined as a distance between suitable patches [see review by 3]. More recently, community structure in dendritic systems, such as streams and rivers, has been placed in a metacommunity framework [4–6].

Trematodes (Phylum: Platyhelminthes, Subclass: Digenea), also known as flukes or parasitic flatworms, are common in aquatic systems. They have complex life cycles, usually involving a series of three hosts, although life histories vary. Adult trematodes sexually reproduce in vertebrate definitive hosts and release eggs into the environment via host feces. Eggs hatch into larvae that infect aquatic mollusks as first-intermediate hosts and reproduce asexually to generate free-swimming cercariae. Cercariae leave mollusks and form metacercarial cysts either in second-intermediate hosts (invertebrate or vertebrate) or on aquatic vegetation. The life cycle is completed when a definitive host consumes an infected second-intermediate host or ingests environmental cysts [7].

For most parasites, including many trematodes, host dispersal is typically greater than that of any free-living parasite stage. Because intermediate hosts are often less mobile than definitive hosts, the structure of larval trematode communities is often highly correlated with the distribution and abundance of definitive hosts [8–10]. Differences in the dispersal abilities of definitive host species can drive variation in patterns of trematode abundance and diversity. For instance, if the definitive host is a fish, a trematode life cycle may be entirely aquatic (autogenic life cycle) and limited to within site dispersal; however, if the definitive host is a terrestrial mammal or bird (allogenic life cycle), trematodes may disperse with their hosts across the landscape [see 11]. A study in a set of inter-connected lakes has demonstrated that these differences in definitive host dispersal can impact trematode distributions across a landscape [12]. Trematode community structure is also determined by local factors, such as land use and water quality, as environmental conditions must be suitable for the co-occurrence of all hosts and free-living larval stages [13–15].

Most research on the processes that drive variation in larval trematode communities has been conducted in ponds or lakes [e.g. 16, 17] or marine systems [8, 10]. However, due to the dendritic structure of stream networks, and the potential effects of continuous downstream current moving infectious stages and aquatic hosts, infection dynamics of parasites in stream systems may differ from those in other aquatic systems [e.g. 18]. For trematode distributions, in particular, recent studies in river and stream systems suggest that downstream flow can be important in determining trematode abundance [e.g. 11]; however, in at least some cases [e.g. 19], local-scale environmental factors may still be more important in determining parasite structure along a river continuum.

Here, we report the results of two studies in stream systems conducted at different spatial scales to examine the factors driving larval trematode community structure. First, we conducted a landscape-level survey to characterize the regional diversity and abundance of trematodes infecting stream snails *Elimia* (= *Oxytrema* = *Goniobasis*) *proxima* (Gastropoda: Pleuroceridae). *Elimia proxima* is a common, native inhabitant of Appalachian headwater streams [20] that serves as first-intermediate host to a number of trematode species, some with autogenic, and some with allogenic, life cycles. Our main objective in the first study was to examine the importance of local and regional processes in shaping patterns of parasite distribution across stream networks. In the second study, we investigated the effects of dendritic network structure on the distribution of larval trematodes within a single stream network. We characterized *E*. *proxima* trematode communities in headwater and main stem sites to determine if there was a downstream gradient of trematode prevalence and diversity, which could result from continuous stream flow moving free-living parasite stages and infected hosts downstream.

A secondary objective of our studies was to assess variation in morphological vs. molecular approaches to quantifying diversity in larval trematode systems. Larval trematode systems are increasingly used to address important ecological theory [e.g. 21, 22], and these studies typically rely on large sample sizes and rapid visual assessment of larval trematodes for species identification. However, most species-level descriptions of trematodes are based on adult worms, which differ significantly from the larval forms. To determine whether morphological assessments were acceptable estimates of diversity, we used both morphological and molecular approaches to define our communities in both our landscape-level and within-stream studies.

## Materials and methods

### Study sites

**Fig 1:**
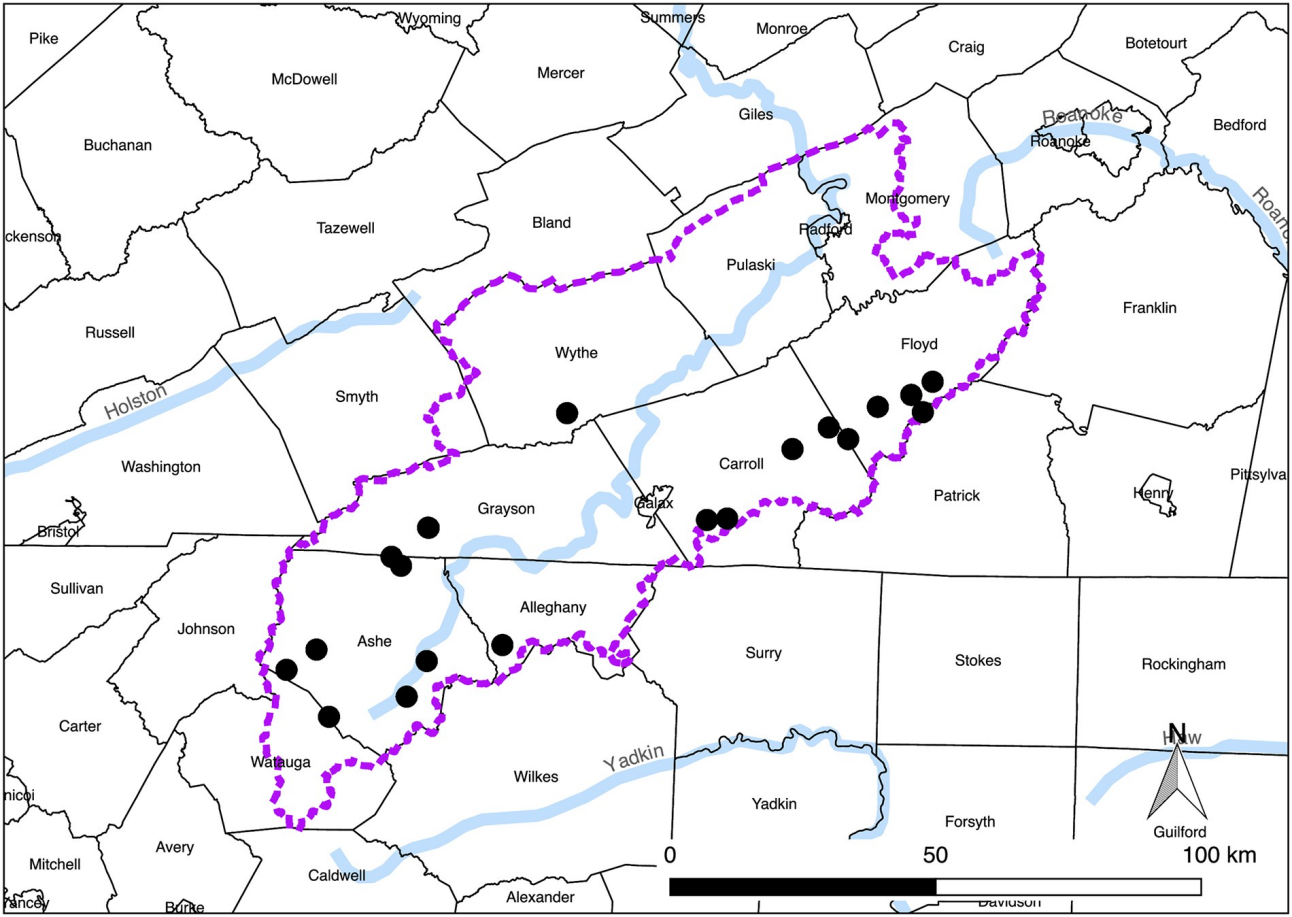
Map of landscape-level study sites. Location of the 20 landscape-level study sites in southwestern Virginia and northwestern North Carolina within the Upper New River Basin (demarcated by dashed purple line).

In the landscape-level study, we examined first-intermediate host trematode infection of *E*. *proxima* in 20 Appalachian headwater streams distributed across four counties in southwestern Virginia and two counties in northwestern North Carolina in summer 2011 (Fig 1, S1 and S2 Tables in S1 Appendix).

**Fig 2:**
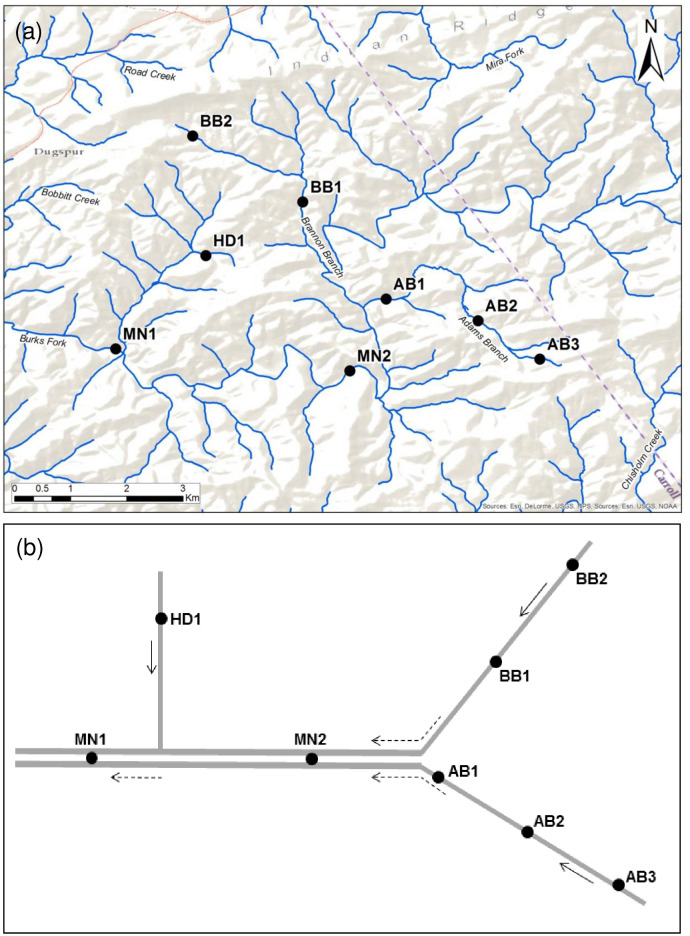
Map and diagram of within-network study sites. (a) Location of eight within-network study sites in the Big Reed Island Creek drainage in Carroll Co., VA, and (b) simplified stream network diagram indicating relative position of sites within network configuration (arrows indicate direction of flow). Main stem sites (MN) and headwater sites (HD, AB, BB).

At one of these sites in Carroll County, VA, we subsequently completed a within-network study in summer 2014 by examining trematode infection of *E*. *proxima* at eight sites within a single stream network. These included two sites in the main stem and six sites in three different headwater streams (Fig 2, S3 and S4 Tables in S1 Appendix). Scientific collection permits were obtained from the Virginia Department of Game and Inland Fisheries (#038862 and #050490).

### Snail density and environmental variables

For the landscape-level study, we established a 50 m study reach at each site and quantified *E*. *proxima* density with a 1/3 m^2^ quadrat sampler placed at a minimum of 15 randomly selected points. We used a handheld meter (YSI Model 63, YSI, Inc., Yellow Springs, OH) to measure pH, specific conductance, and water temperature. We also collected water samples to measure phosphorous and nitrogen concentrations because higher nutrient levels could contribute to higher snail densities and increased trematode infection [14]. Total phosphorous and total nitrogen were quantified with standard colorimetric assays [23] using a Lachat flow-injection autoanalyzer (Hach Company, Loveland, CO).

We followed the same methods for the within-network study, but we increased quadrat sampling to measure snail density at 30 points, and because no snails < 0.05 g from the landscape-level study were infected, snails < 0.05 g were not included in density measurements. We also measured dissolved oxygen (YSI Model 550A, YSI, Inc., Yellow Springs, OH) and estimated stream discharge. Nutrient levels were not measured for the within-network study.

### Trematode collection

At each site, we haphazardly hand-collected a sample of ~120 *E*. *proxima* for trematode screening (N = 2,515 snails comprised of all sizes for landscape-level study; N = 946 snails > 0.05 g for within-network study). In the laboratory, we recorded the wet mass of each snail, and used a dissecting microscope to examine the gonadal tissue and digestive tract for larval trematodes (sporocysts, rediae and cercariae). We identified trematode infections as one of five morphotypes based on the morphology of cercariae described in Schell [24] and primary sources: (1) *Metagonimoides oregonensis* (pleurolophocercous cercariae previously identified in *E*. *proxima* in [25, 26]); (2) *Sanguinicola* sp. [27, 28]; (3) virgulate [29–31]; (4) cotylomicrocercous [32, 33]; and (5) monostome [34] (Table 1). For the within-network study, we further categorized the virgulate-type cercariae as "small" or "large" morphotypes because after examining so many infected snails for the preceding study, it became apparent that there were at least two types of virgulate cercariae (Table 1). From every infected snail, we also preserved larval trematode samples in 95% ethanol at -20°C for molecular identification.

**Table 1:** Larval trematodes infecting *E*. *proxima* as a first-intermediate host across 20 sites in the landscape-level study and eight sites in the within-network study, including the results of BLAST searches (conducted May 5, 2016) of each haplotype identified via molecular analysis.

	Potential hosts*		No. of sites	Prev (%)	No. of haplotypes	No. of samples	Matches to GenBank sequences		
Morphotype of cercariae	Second intermediate	Definitive					Species (Max Identity %)	Accession No.	Family(s)
Cotylomicrocercous	Aquatic insects	Fish	17	0–38	1	119	*Plagiocirrus loboides* (99)	EF523477.1	Opecoelidae
					6	45	*Plagiocirrus loboides* (98)	EF523477.1	Opecoelidae
					2	5	*Plagiocirrus loboides* (97)	EF523477.1	Opecoelidae
*Metagonimoides oregonensis*	Amphibians	Raccoons, mink	20	2–20	1	70	*Metagonimoides oregonensis* (99)	JQ995473.1	Heterophyidae
					5	117	*Metagonimoides oregonensis* (98)	JQ995473.1	Heterophyidae
	Fish	Piscivorous mammals			1	5	*Clonorchis sinensis* (97)	JF823989.1	Opisthorchiidae
Virgulate	Aquatic insects	Bats, birds	20	< 1–10	3	28	*Paralecithodendrium parvouterus* (97)	AY220617.1	Lecithodendriidae
					1	7	*Lecithodendrium linstowi* (97)	AF151919.1	Lecithodendriidae
					2	29	*Collyriclum faba* (96)	JQ231122.1	Collyriclidae
					1	6	*Allassogonoporus amphoraeformis* (93)	AF151924.1	Pleurogenidae
					1	38	*Allassogonoporus amphoraeformis* (92)	AF151924.1	Pleurogenidae
*Sanguinicola* sp.	None	Fish	3	0–6	1	8	*Sanguinicola* cf. inermis (85)	AY222180.1	Aporocotylidae
Monostome	None; encysts on vegetation	Birds (anseriforms)	1	0–4	1	5	*Notocotylus* sp. BH-2008 (98)	EU712725.1	Notocotylidae
Immature or unknown	variable	variable			1	1	Echinostomatidae sp. 1 (99)	GU270100.1	Echinostomatidae
					1	2	*Plagiocirrus loboides* (97)	EF523477.1	Opecoelidae
					1	5	*Collyricloides massanae* (94)	KP682451.1	Pleurogenidae
					1	1	*Parabascus duboisi* (94)	AY220618.1	Pleurogenidae

### Molecular identification of trematodes

Larval trematodes frequently lack the distinguishing morphological features of adult worms that are used for visual identification. Molecular identification of larval forms is often necessary, especially in preliminary research of new study systems, to adequately capture parasite diversity. To confirm visual identification and examine genetic variation within morphotypes, we sequenced ~1,400 bp of the 28S large subunit rRNA gene [following 35] with modifications (see S1 Appendix). For each infected snail from the landscape-level study (N = 548, excluding 13 snails with dual infections N = 535), DNA was extracted from a single sporocyst or redia, or when no other parasite tissue was available, we pooled ~ 5 cercariae. Sequences were assigned to haplotype, differentiated by the presence of any single-nucleotide polymorphism, and compared to trematode sequences in GenBank via BLAST search. Sequences of each haplotype were deposited in the NCBI GenBank (Accession numbers MH094412 –MH094439).

To examine genetic variation within a single stream network, we used the same methods described above to obtain partial 28S rRNA gene sequences for trematodes from each infected snail (N = 284). These sequences were matched to the haplotypes established from the landscape-level study; however, because sequence quality was generally lower for the samples from this study, we identified fewer unique haplotypes within each morphotype.

### Statistical analysis

#### Landscape-level study

To visualize relationships among sites based on trematode community composition, we conducted principal coordinates analysis (PCoA) of trematode prevalence using Bray-Curtis dissimilarity. We conducted distance-based redundancy analysis (db-RDA) to test for both species-environment relationships and spatial patterns in trematode community composition. The environmental variables we examined included *E*. *proxima* density, stream width, pH, specific conductance, total nitrogen, total phosphorous and elevation. To create spatial variables, we used principal coordinates of neighbor matrices (PCNM) [36, 37] to extract eigenvectors from geographic coordinates. PCNM eigenvectors represent a range of broad to fine scale spatial patterns. The first PCNM eigenvector (PCNM1) represents the broadest scale spatial pattern, a linear pattern. Additional PCNM eigenvectors represent successively finer scale (more complex) spatial patterns, such as patches or gaps [38]. For variable selection in both spatial and environmental db-RDA models, we used forward stepwise model selection based on adjusted R^2^ values [39]. With the selected spatial and environmental variables, we then conducted variation partitioning to determine the proportion of across site community variation explained solely by environmental variables, solely by spatial variables, or by spatially-structured environmental variables (i.e. neither solely environmental or spatial) [38]. To determine if community patterns remained the same when examined at different levels of taxonomic resolution, all analyses were conducted for both morphotype (based on visual identification) and haplotype (based on molecular identification) defined communities. For this dataset, we also examined whether there was a correlation between richness estimates for each site based on morphotype and haplotype datasets. A lack of a correlation could indicate that morphologically-based diversity estimates are unlikely to provide a true picture of larval parasite communities. All analyses were conducted using the *labdsv* [40] and *vegan* [41] packages in R v. 3.2.4 [42].

#### Within-network study

To test for a downstream gradient of infection, we used binomial generalized linear models (GLMs) to model the relationship between trematode prevalence and in-stream distance from the main stem site located farthest downstream (MN1). We also used binomial GLMs to examine species-environment relationships. Total trematode prevalence, as well as the prevalence of each morphotype, was modelled separately to determine if infection patterns differed for allogenic trematodes (*M*. *oregonensis*, small and large virgulate types) versus autogenic trematodes (cotylomicrocercous type). Predictor variables for species-environment relationship models included *E*. *proxima* density, pH, specific conductance, dissolved oxygen, stream depth and site elevation. Because stream depth and width were highly correlated, we did not include both parameters. Additionally, because snail mass in field surveys could represent both an explanatory and a response variable, we did not include snail mass in models. While larger, older snails may be more likely to be infected because of increased potential exposure time, trematode infected snails can exhibit gigantism as a result of castration due to heavy infections in the gonadal tissue [43, 44]. For GLM selection, we chose best subsets of full models based on AICc or QAICc.

To examine relationships between site-level factors and variation in trematode community composition, we conducted redundancy analysis (RDA) on Hellinger transformed morphotype prevalence. Site-level factors included snail density and all abiotic variables measured. We used forward stepwise model selection based on adjusted R^2^ values. To assess the relationship between spatial distribution and variation in trematode community composition, we conducted an additional RDA with the predictor variables Euclidean and in-stream distance from the main stem site located farthest downstream (MN1). We used both distances because Euclidean distance is an overland distance that might be more relevant for allogenic parasites, while in-stream distance is likely more relevant for autogenic parasites limited to in-stream dispersal. Because within-network haplotype diversity was low relative to the number of morphotypes, we did not conduct additional tests for haplotype-defined communities (Table 1). All analyses were conducted in R v. 3.2.4 [42] using the *AICcmodavg* [45] and *vegan* [41] packages.

## Results

### Trematode prevalence and diversity

#### Landscape-level study

**Fig 3:**
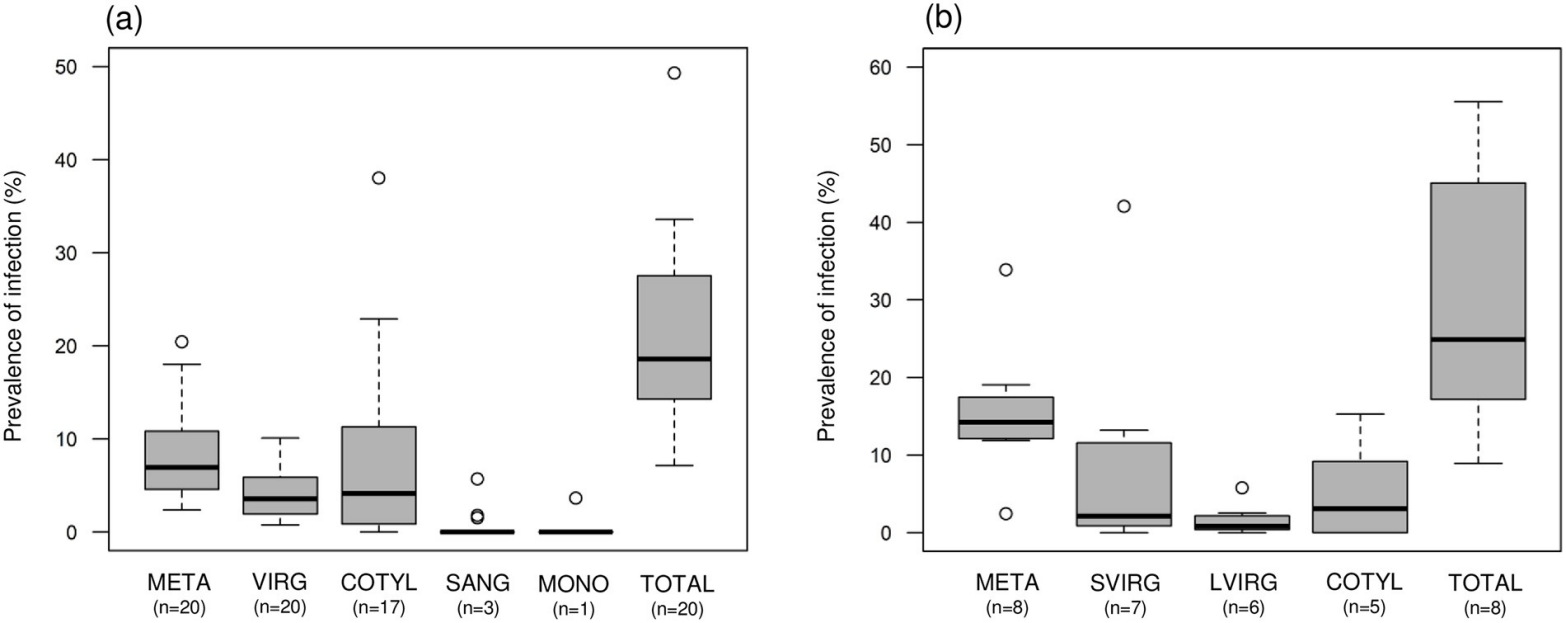
Box and whisker plot of trematode morphotype prevalence. Median and range of prevalence of each trematode morphotype across (a) all 20 landscape-level study sites and (b) all 8 within-network sites. Whiskers represent 1.5 interquartile range. Trematode morphotypes: *Metagonimoides oregonensis* (META); virgulate (VIRG); cotylomicrocercous type (COTYL); *Sanguinicola* sp. (SANG); and monostome type (MONO). In the within-network study (b) virgulate infections were categorized as small virgulate (SVIRG) and large virgulate (LVIRG).

**Fig 4:**
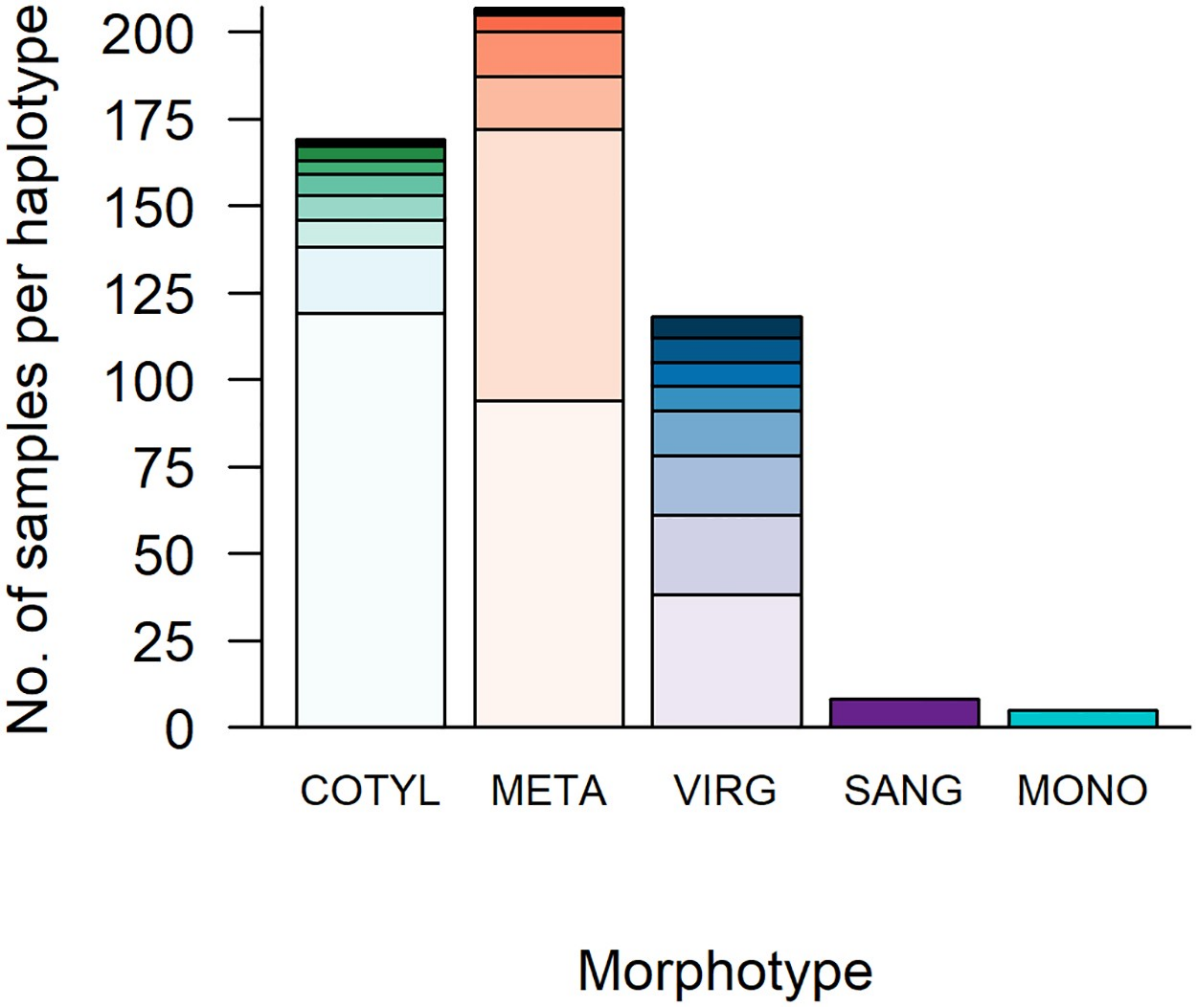
Stacked bar chart displaying the number and relative proportion of unique haplotypes within each morphotype. From the landscape-level study, the number of trematode samples (total N = 491 samples) per unique haplotype identified within each of the five morphotypes. Abbreviations and total number of unique haplotypes identified for each morphotype are as follows: cotylomicrocercous type (COTYL; n = 9 haplotypes); *Metagonimoides oregonensis* (META; n = 7 haplotypes); virgulate (VIRG; n = 8 haplotypes); *Sanguinicola* sp. (SANG; n = 1 haplotype); and monostome type (MONO; n = 1 haplotype). Different colors within bars show the proportion of samples per unique haplotype.

Out of 2,515 *E*. *proxima* screened, 548 snails were infected with trematodes. Trematodes were present at all sites, and total prevalence of infection ranged from 10% (Chisholm Creek) to 49% (Little Wilson Creek) (Fig 3a, S1 Table in S1 Appendix). Both *M*. *oregonensis* and virgulate infections were encountered at all 20 sites. We obtained partial 28S rRNA gene sequences for 491 larval trematodes, from which we identified 30 unique haplotypes belonging to 9 families (Table 1, Fig 4, S1 Appendix). For most morphotypes, 1 to 2 haplotypes comprised >80% of all infections (Fig 4). We did not find a significant correlation between the morphotype and haplotype-based estimates of richness for each site (r = 0.155, p-value = 0.514).

#### Within-network study

Out of 946 *E*. *proxima* examined, 284 snails were infected. Total prevalence of infection ranged from 8.9% (MN1) to 55.6% of snails infected (ADB3) (Fig 3b; S2 Table in S1 Appendix). *Metagonimoides oregonensis* was present at all eight sites and ranged in prevalence from 2.4% (MN1) to 33.9% (BB2). We obtained partial 28S rRNA gene sequences for 227 larval trematodes from which we identified seven unique haplotypes belonging to four families (Table 1, S1 Appendix).

### Community variation and spatial distribution

#### Landscape-level study

**Fig 5:**
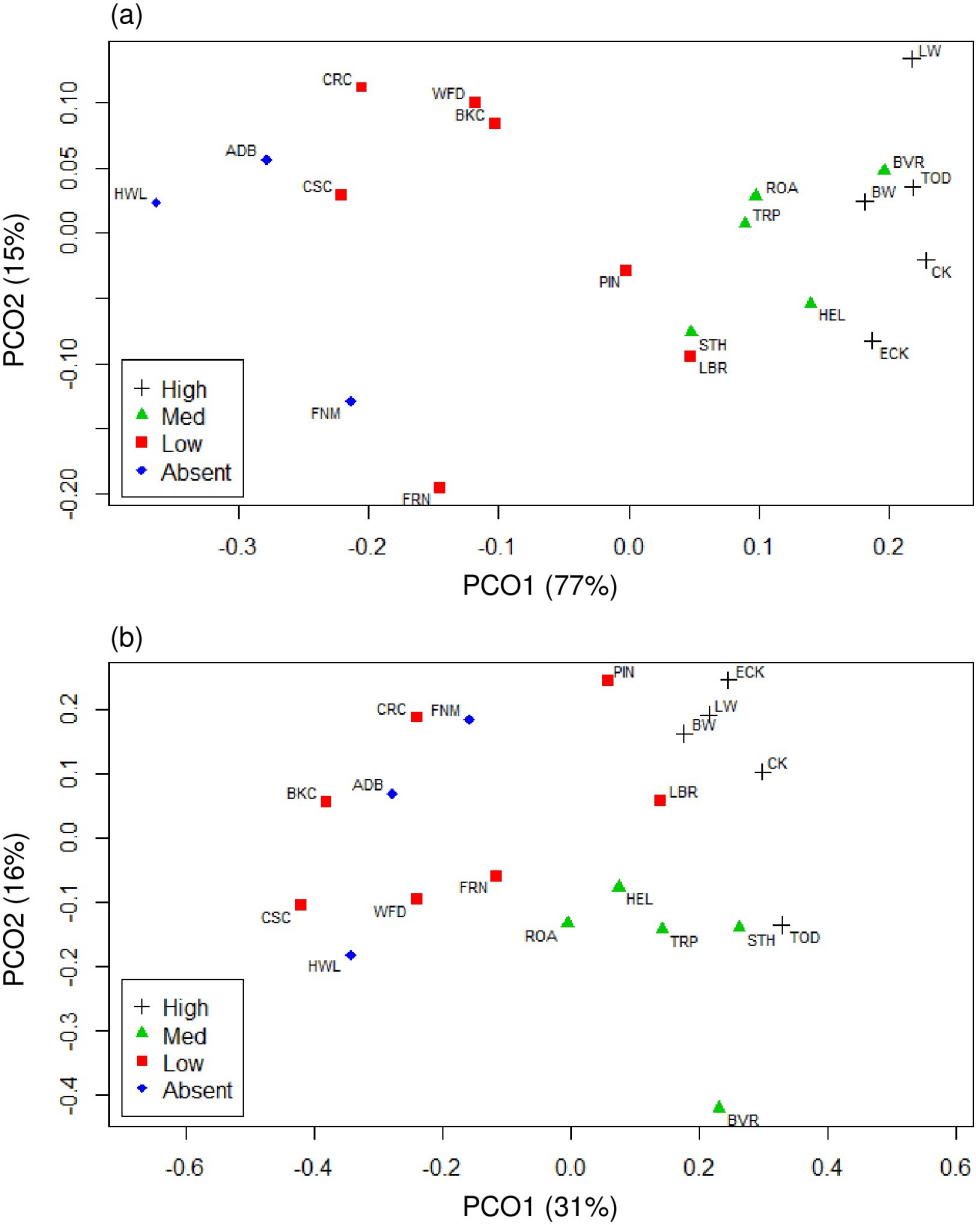
Plots of PCoA axes for landscape-level study morphotype and haplotype communities. PCoA of Bray-Curtis dissimilarity from landscape-level study for (a) morphotype defined communities and (b) haplotype defined communities. Percent variance explained by each principal coordinate included in axis label. Sites are labeled with abbreviations (see S1 Table in S1 Appendix) and categorized by prevalence of cotylomicrocercous type infections defined as: High > 10%; Medium = 5–10%; and Low < 5% of snails infected. “None” indicates absence of cotylomicrocercous type.

*Metagonimoides oregonensis* was the most prevalent parasite at 11 of the 20 sites, while the cotylomicrocercous type was most prevalent at 9 sites (S1 Table). The prevalence of these two types was highly negatively correlated (r = -0.897, p < 0.001), and PCoA ordinations of morphotype and haplotype prevalence showed that variation in trematode communities was driven primarily by these dominant species (Fig 5). For PCoA of morphotype-defined communities, the first two principal coordinates accounted for 92% of the variance in trematode communities. The first principal coordinate accounted for 77% of the total variance, and was most correlated with cotylomicrocercous type species (r = 0.97) and *M*. *oregonensis* (r = -0.95). The second principal coordinate accounted for an additional 15% of the total variance, and was most correlated with virgulate type species (r = -0.86).

In PCoA of haplotype-defined communities, a greater number of principal coordinates was needed to capture the variance in composition. For PCoA of haplotype communities, the first two principal coordinates accounted for 47% of the variance in trematode communities. The first principal coordinate accounted for 31% of the total variance, and was most correlated with a cotylomicrocercous haplotype (r = 0.75) and a *M*. *oregonensis* haplotype (r = -0.74). The second principal coordinate accounted for an additional 16% of the total variance, and was most correlated with a virgulate haplotype (r = 0.71).

From PCNM analysis, we obtained 12 positive eigenvectors used as spatial variables in db-RDA to test for spatial structuring of trematode communities. Trematode communities defined by morphotypes were spatially structured at the broadest scale (PCNM1) based both on prevalence (Bray-Curtis: adj. R^2^ = 0.215, p = 0.003) and presence-absence of morphotypes (Jaccard: adj. R^2^ = 0.243, p = 0.002) (S3 Table, S1 Fig in S1 Appendix). PCNM1 was positively correlated with cotylomicrocercous morphotype prevalence (r = 0.673, p = 0.001) and negatively correlated with *M*. *oregonensis* morphotype prevalence (r = -0.627, p = 0.003) (S1 Fig in S1 Appendix). Trematode communities defined by haplotypes were also structured at the broadest spatial scale (PCNM1) and related to additional broad scale spatial variables (PCNM2 and PCNM3) based on haplotype prevalence (Bray-Curtis: adj. R^2^ = 0.199, p = 0.001) and presence-absence of haplotypes (Jaccard: adj. R^2^ = 0.206, p = 0.001) (S3 Table, S1 Fig in S1 Appendix).

#### Within-network study

Trematode community composition varied across sites. The trematode communities of the headwater sites located farthest upstream (AB3 and BB2), as well as another headwater site (HD1), were dominated by either *M*. *oregonensis* or small virgulate infections, whereas cotylomicrocercous infections were completely absent. Cotylomicrocercous infections were most prevalent at sites with intermediate depth, 6.4 to 7.7 cm (BB1 and AB2), and comprised the majority of infections at the main stem site located farthest downstream (MN1). In RDA, neither geographic distance metric (Euclidean or in-stream) significantly explained the variation in trematode communities.

Total prevalence of infection decreased from headwater to main stem sites (S2 Table in S1 Appendix), and there was a significant positive relationship between total prevalence of infection and in-stream distance from headwater to main stem (β = 0.185, SE = 0.033, p = 0.001). The same pattern was observed qualitatively for both *M*. *oregonensis* and small virgulate infections, but no relationships with in-stream distance for any of the individual morphotypes were statistically significant.

### Environmental heterogeneity and relationships with local factors

#### Landscape-level study

Snail density and water quality varied across sites (S1 Table in S1 Appendix). There were no significant relationships between any of the measured environmental variables and variation in morphotype communities. While elevation was positively correlated with cotylomicrocercous type prevalence (r = 0.473, p = 0.035), it was not significant in db-RDA of morphotype communities. For both dissimilarity metrics, variation in haplotype communities was significantly related to elevation. Elevation explained only a small proportion of the total variation (adj. R^2^ = 0.05, S3 Table in S1 Appendix) and was not independent of spatial variation, as elevation was positively correlated with PCNM1 (r = 0.50, p = 0.026). No other environmental variables were significantly related to haplotype community variation.

#### Within-network study

Elevation decreased from headwater to main stem sites, concurrent with increased stream width, depth and discharge (S2 Table in S1 Appendix). Dissolved oxygen at all sites was at or near saturation and did not differ appreciably across sites. Mean snail density ranged from 0.8 (± 0.4) snails/m^2^ at main stem site MN1 up to 46.1 (± 8.0) snails/m^2^ at headwater site BB2 (S2 Table in S1 Appendix). Snail density co-varied positively with elevation and negatively with stream width, depth, and discharge. Snail density was most strongly correlated with stream width (adj. R^2^ = 0.92, p = 0.0001).

Total prevalence of infection was not significantly related to snail density. The optimal GLM of total prevalence of infection included only elevation as an explanatory variable (Akaike weight = 0.95, β = 0.012, SE = 0.002 ⅇ^β^ = 1.012). The prevalence of *M*. *oregonensis* was positively related to snail density (Akaike weight = 0.5, β = 0.034, SE = 0.009, ⅇ^β^ = 1.035) and negatively related to mean maximum depth (Akaike weight = 0.33, β = -0.09, SE = 0.033, ⅇ^β^ = 0.914). Small virgulate prevalence was positively related to elevation (Akaike weight = 0.38, β = 0.015, SE = 0.006, ⅇ^β^ = 1.015) and negatively related to conductivity (Akaike weight = 0.38, β = -0.05, SE = 0.026, ⅇ^β^ = 0.947). For both the large virgulate and cotylomicrocercous types, there were no significant relationships with snail density or any of the environmental factors measured. In RDA, elevation was the only environmental variable that significantly explained variation in trematode communities (adj. R^2^ = 0.359, p = 0.022).

## Discussion

At the landscape level, we found that trematode communities exhibited a broad scale spatial pattern due to regional turnover in the dominant trematode species for both morphotype- and haplotype-defined community structure. Sites in the southwestern part of the study area were characterized by high prevalence of cotylomicrocercous type infections. Conversely, cotylomicrocercous type infections were either absent or had relatively low prevalence at sites located in the northeastern part of the study region, where the prevalence of *M*. *oregonensis* was high. This spatial pattern was correlated with elevation, but elevation was a significant predictor of community variation only for haplotype-defined communities, suggesting that the significance of elevation emerged as a result of capturing additional heterogeneity within communities by using haplotype-level resolution. The relationship with elevation could be related to dispersal differences between autogenic and allogenic parasites, and/or could be due to the aggregate effect of local factors correlated with elevation (e.g. land use, canopy cover, flow regime).

We did not find evidence that variation in community structure across the landscape (from 20 headwater streams spanning ~130 km) was related to snail density or local environmental factors. It was somewhat surprising that we did not see a link with nitrogen and phosphorous levels, as this contrasts with the results of previous studies in pond and lake systems, where nutrient inputs, and resulting eutrophication led to increases in snail biomass and trematode infection [14, 15]. One explanation for this contrast is differences in habitat requirements of first-intermediate hosts in pond systems; pulmonate (lung-breathing) snails thrive in environments with high levels of primary production resulting from increased nutrient inputs, despite subsequent oxygen depletion. The gastropod in our host-parasite system, *E*. *proxima*, is a prosobranch (gill-breathing) snail adapted to high levels of dissolved oxygen, as found in headwater streams, and we might not expect populations to persist at sites that are highly impacted by eutrophication. Local factors beyond nutrient inputs and eutrophication can also be important in river systems. Blanar et al. [19] found fish parasite community structure in a river was most strongly driven by local factors, namely sediment hydrocarbons and local land use around the site. However, we did not find that to be the case in our study, suggesting that broader landscape-level factors, such as dispersal, likely drive community structure across streams.

Within a stream network, we found relationships between trematode infection and local factors for two of the four trematode types. *Metagonimoides oregonensis* was positively related to snail density and negatively related to stream depth. While a positive relationship between host density and infection is often expected for parasites with direct transmission, for complex life cycle parasites, such as trematodes, the relationship is often less clear [46]. Previous research in freshwater trematode systems has found positive [14, 47], negative [48], and no association [13, 49] between snail infection and snail density. For *M*. *oregonensis*, a positive relationship with snail density suggests that other factors affecting prevalence are not limiting snail infection. Namely, that egg input from raccoon definitive hosts is high, salamander second-intermediate hosts are abundant, and stream flow is conducive to transmission of miracidial and cercarial stages. For the small virgulate morphotype, prevalence was negatively related to conductivity, which could be due to a reduction in suitable aquatic insect second-intermediate hosts as stream order and conductivity increase. Small virgulate prevalence was also positively related to elevation, but this relationship is more difficult to interpret, because many environmental factors (e.g. canopy cover, water temperature, stream flow) correlate with elevation. A larger survey that incorporates sites across a wider range of environmental conditions and considers additional factors, like land use, would be necessary to assess these correlations more completely.

Blasco-Costa et al. [11] generally found increased abundances of fish trematode parasites downstream when surveying along a river gradient. We expected we might find that as well because of continuous flow pushing trematode larval stages and snail hosts downstream. However, we did not find an increasing downstream gradient of infection prevalence within streams. Instead we observed the opposite pattern, with the highest trematode prevalence in the headwater sites farthest upstream. This pattern was significant only for total prevalence of infection, but we observed this same pattern for *M*. *oregonensis* and small virgulate infections. Cotylomicrocercous infections exhibited a non-linear pattern of distribution. Cotylomicrocercous trematodes were not present at the headwater sites farthest upstream, presumably because the stream depth was not adequate for fish definitive hosts. These parasites reached highest prevalence at intermediate headwater sites and remained present in main stem sites. This suggests that sites with intermediate stream depth are the sites of greatest overlap between the aquatic insect second-intermediate hosts and fish definitive hosts for this parasite; this could be tested with more targeted sampling in the future. The prevalence of large virgulate infections was generally low, and we did not observe a clear spatial pattern in the prevalence of this type within the network.

We did find that diversity in headwater streams consisted of nested subsets of main stem communities. If *E*. *proxima* found inhabiting main stem sites are from source populations upstream, main stem diversity could be due to the effects of downstream drift; a population genetics approach might be able to more clearly address this question [e.g. 50]. These results are congruent with metacommunity studies of stream insects, in which species sorting according to local environmental factors seems to largely counteract the effects of downstream drift, especially in headwater streams, and to a higher degree for species with terrestrial adults [4, 5, 51].

Unresolved cryptic diversity can confound our understanding of parasite ecology and evolution, because genetically distinct species may differ in host use, pathology, site-specificity and dispersal. For example, molecular surveys of parasitoids indicate that species considered "generalists" can actually be suites of cryptic "specialists" [52], which could confound attempts to use parasitoids for biological control of insect pests, and genotypic variation in tapeworms can cause differences in developmental rate and virulence that impact disease control strategies [53]. Molecular studies of larval trematodes have also revealed that cryptic species diversity can obscure patterns of spatial distribution and host specificity [54–57]. For instance, molecular analysis of a geographically wide-spread trematode revealed a complex of eight cryptic (morphologically indistinguishable) trematode species, some with very limited geographic ranges [58]. As we increasingly rely on rapid morphological assessments to assign larval trematodes to species and use these estimates to test ecological theory, it is important to understand whether our estimates accurately represent the system. We did not find a significant correlation between morphotype richness and haplotype richness, which further suggests that molecular analysis of larval trematodes may be important for research conducted in these systems.

The D1- D3 region of the large subunit rRNA (28S) gene is often used as a marker to resolve phylogenies [35, 59] and identify cryptic species within Digenea; sequence divergence at the 28S rRNA gene as low as 0.4% [60, 61] or 0.8% [62] is evidence of speciation. Based on the divergence of haplotypes we observed, we conclude that there is support for the existence of multiple species within each of the three larval morphotypes (cotylomicrocercous type, *M*. *oregonensis* and virgulate type) that contained multiple haplotypes. Pairwise sequence divergence of 0.7%–4.0% between eight of nine cotylomicrocercous haplotypes suggests this morphotype may have comprised up to eight species in our study. For the seven haplotypes identified from *M*. *oregonensis* infections, sequence divergence of 1.0–1.3% suggests the presence of at least two unique species within Heterophyidae, plus a third species from Opisthorchiidae (6.4–6.7% divergence from heterophyid haplotypes). Finally, sequence divergence between virgulate haplotypes, suggests this morphotype may comprise up to five species: one species most closely related to Collyriclidae, one species most closely related to Pleurogenidae, and an additional three species within Lecithodendriidae (sequence divergence of 0.5–5.2%). Further support for species delimitation could be provided by molecular analysis at additional loci, and more detailed morphological analysis is necessary to confirm cryptic speciation [63].

Morphotypes differed in both the level of sequence divergence among respective haplotypes, and in the distribution of individual haplotypes across the study region. This may reflect differences in the dispersal abilities of definitive host species. We found the highest level of genetic diversity within the cotylomicrocercous morphotype, potentially representing up to eight species, and four of these were found at only a single site. These trematodes have autogenic life cycles, with fish definitive hosts that have different dispersal constraints than terrestrial hosts. These constraints can result in greater geographic isolation and higher genetic diversification [64–67]. For example, Criscione and Blouin [65] found that autogenic trematode species exhibited less gene flow and had more geographically-structured populations than allogenic species. In our study, there was less sequence divergence among the six heterophyid haplotypes from the allogenic *M*. *oregonensis* morphotype, which may comprise two species. These allogenic parasites may disperse across the landscape with their terrestrial hosts (raccoon and mink), and this may also explain the much broader distribution of these haplotypes across the study region. Note, two of these six haplotypes were present at only a single site, but their low divergence from other haplotypes does not suggest speciation. Finally, three of the four lecithodendriid haplotypes within the virgulate morphotype may represent unique species, and one of these haplotypes was encountered at a single site, while the others were more broadly distributed. These are also allogenic parasites, most likely with bats or birds as definitive hosts, so we would expect these trematodes to be broadly distributed, but not necessarily to show a high degree of speciation. The difference in genetic diversity between the two allogenic morphotypes may reflect a greater diversity in definitive host species (i.e. many species of bats and birds) among the lecithodendriid trematodes, versus two potential definitive host species, raccoons and mink, in the heterophyids.

By using a metacommunity framework that considers how species disperse within and across stream networks, in addition to how local factors impact species abundance, this research advances our understanding of the processes that structure communities in streams. We found a broad scale compositional shift across the landscape from communities dominated by allogenic parasites (e.g. raccoons) to communities dominated by autogenic parasites (e.g. fish). Allogenic parasites were broadly distributed across the study region, while many autogenic parasites were more geographically limited, which may reflect constraints on fish dispersal within stream networks and correspondingly, across the landscape. While spatial variation in communities was correlated with elevation, we did not find significant relationships with any other local factors investigated at the landscape-level. In contrast, within a stream network, several local factors were significant, suggesting that community structure is driven by heterogeneity in local factors. The decreasing downstream gradient of infection from headwaters to main stem suggests that site-level factors largely countered the effects of downstream dispersal, as has been described in other stream taxa. Additionally, we found that each trematode morphotype potentially comprises multiple species, and that genetic diversity within morphotypes may reflect dispersal abilities related to allogenic versus autogenic life cycles. If each morphotype comprises multiple species, incorporating this information in future research could elucidate relationships that may be masked by the lumping of morphologically-similar species, especially for investigations conducted at larger spatial scales.

## Supporting information

S1 Appendix Additional methods and results. (DOCX)

## References

[1] Holyoak M, Leibold MA, Holt RD, editors. Metacommunities: Spatial Dynamics and Ecological Communities. Chicago, IL: University of Chicago Press; 2005.

[2] Leibold MA, Holyoak M, Mouquet N, Amarasekare P, Chase JM, Hoopes MF, et al The metacommunity concept: a framework for multi-scale community ecology. Ecol Lett. 2004;7: 601–613. 10.1111/j.1461-0248.2004.00608.x

[3] Logue JB, Mouquet N, Peter H, Hillebrand H. Empirical approaches to metacommunities: a review and comparison with theory. Trends Ecol Evol. 2011;26: 482–491. 10.1016/j.tree.2011.04.009 21641673

[4] Brown BL, Swan CM. Dendritic network structure constrains metacommunity properties in riverine ecosystems. J Anim Ecol. 2010;79: 571–80. 10.1111/j.1365-2656.2010.01668.x 20180874

[5] Grönroos M, Heino J, Siqueira T, Landeiro VL, Kotanen J, Bini LM. Metacommunity structuring in stream networks: roles of dispersal mode, distance type, and regional environmental context. Ecol Evol. 2013;3: 4473–4487. 10.1002/ece3.834 24340188 PMC3856747

[6] Tonkin JD, Heino J, Altermatt F. Metacommunities in river networks: The importance of network structure and connectivity on patterns and processes. Freshw Biol. 2018;63: 1–5. 10.1111/fwb.13045

[7] Bush AO, Fernandez JC, Esch GW, Seed JR. Platyhelminthes: the flatworms Parasitism: The diversity and ecology of animal parasites. Cambridge, UK: Cambridge University Press; 2001 pp. 103–159.

[8] Byers JE, Blakeslee AMH, Linder E, Cooper AB, Maguire TJ. Controls of spatial variation in the prevalence of trematode parasites infecting a marine snail. Ecology. 2008;89: 439–451. 10.1890/06-1036.1 18409433

[9] Hechinger RF, Lafferty KD. Host diversity begets parasite diversity: bird final hosts and trematodes in snail intermediate hosts. Proc R Soc B Biol Sci. 2005;272: 1059–1066. 10.1098/rspb.2005.3070 16024365 PMC1599879

[10] Levakin IA, Nikolaev KE, Galaktionov K V. Long-term variation in trematode (Trematoda, Digenea) component communities associated with intertidal gastropods is linked to abundance of final hosts. Hydrobiologia. 2013;706: 103–118. 10.1007/s10750-012-1267-x

[11] Blasco-Costa I, Koehler AV, Martin A, Poulin R. Upstream-downstream gradient in infection levels by fish parasites: a common river pattern? Parasitology. 2013;140: 266–274. 10.1017/S0031182012001527 23058079

[12] Paterson RA, Knudsen R, Blasco-Costa I, Dunn AM, Hytterød S, Hansen H. Determinants of parasite distribution in Arctic charr populations: catchment structure versus dispersal potential. J Helminthol. 2019;93: 559–566. 10.1017/S0022149X18000482 29911512

[13] Ciparis S, Iwanowicz DD, Voshell JR. Relationships between nutrient enrichment, pleurocerid snail density and trematode infection rate in streams. Freshw Biol. 2013;58: 1392–1404. 10.1111/fwb.12135

[14] Johnson PTJ, Chase JM, Dosch KL, Hartson RB, Gross JA, Larson DJ, et al Aquatic eutrophication promotes pathogenic infection in amphibians. Proc Natl Acad Sci U S A. 2007;104: 15781–15786. 10.1073/pnas.0707763104 17893332 PMC2000446

[15] Richgels KLD, Hoverman JT, Johnson PTJ. Evaluating the role of regional and local processes in structuring a larval trematode metacommunity of *Helisoma trivolvis*. Ecography (Cop). 2013;36: 854–863. 10.1111/j.1600-0587.2013.07868.x

[16] Faltýnková A, Valtonen ET, Karvonen A. Spatial and temporal structure of the trematode component community in *Valvata macrostoma* (Gastropoda, Prosobranchia). Parasitology. 2008;135: 1691–1699. 10.1017/S0031182008005027 18992180

[17] Soldánová M, Kuris AM, Scholz T, Lafferty KD. The role of spatial and temporal heterogeneity and competition in structuring trematode communities in the great pond snail, *Lymnaea stagnalis* (L.). J Parasitol. 2012;98: 460–71. 10.1645/GE-2964.1 22191581

[18] Carraro L, Mari L, Gatto M, Rinaldo A, Bertuzzo E. Spread of proliferative kidney disease in fish along stream networks: A spatial metacommunity framework. Freshw Biol. 2018;63: 114–127. 10.1111/fwb.12939

[19] Blanar CA, Hewitt M, McMaster M, Kirk J, Wang Z, Norwood W, et al Parasite community similarity in Athabasca River trout-perch (*Percopsis omiscomaycus*) varies with local-scale land use and sediment hydrocarbons, but not distance or linear gradients. Parasitol Res. 2016;115: 3853–3866. 10.1007/s00436-016-5151-x 27314231

[20] Dillon RT. The Ecology of Freshwater Molluscs. Cambridge, UK: Cambridge University Press; 2000.

[21] Johnson PT, Preston DL, Hoverman JT, LaFonte BE. Host and parasite diversity jointly control disease risk in complex communities. Proc Natl Acad Sci. 2013;110: 16916–16921. 10.1073/pnas.1310557110 24082092 PMC3800997

[22] Lafferty KD, Dobson AP, Kuris AM. Parasites dominate food web links. Proc Natl Acad Sci U S A. 2006;103: 11211–11216. 10.1073/pnas.0604755103 16844774 PMC1544067

[23] American Public Health Association. Persulfate Method for Simultaneous Determination of Total Nitrogen and Total Phosphorus 21st ed Eaton A, Clesceri L, Rice E, Greenberg A, editors. Standard Methods for the Examination of Water and Wastewater. 2005.

[24] Schell SC. Handbook of trematodes of North America North of Mexico. Moscow, Idaho: University Press of Idaho; 1985.

[25] Lang BZ, Gleason LN. Life cycle of *Metagonimoides oregonensis* Price, 1931 (Trematoda: Heterophyidae) in North Carolina. J Parasitol. 1967;53: 93.

[26] Belden LK, Peterman WE, Smith SA, Brooks LR, Benfield EF, Black WP, et al *Metagonimoides oregonensis* (Heterophyidae: Digenea) infection in Pleurocerid snails and *Desmognathus quadramaculatus* salamander larvae in Southern Appalachian streams. J Parasitol. 2012;98: 760–767. 10.1645/GE-2986.1 22394058

[27] Hoffman GL, Fried B, Harvey JE. *Sanguinicola fontinalis* sp. nov. (Digenea: Sanguinicolidae): a blood parasite of brook trout, *Salvelinus fontinalis* (Mitchell), and longnose dace, *Rhinichthys cataractae* (Valenciennes). J Fish Dis. 1985;8: 529–538.

[28] Meade TG, Pratt I. Description and life history of *Cardicola alseae* (Trematoda: Sanguinicolidae). J Parasitol. 1965;51: 575–578. 14339369

[29] Hall JE. Studies on the life history of *Mosesia chordeilesia* Mcmullen, 1936 (Trematoda: Lecithodendriidae). J Parasitol. 1959;45: 327–336. 13665473

[30] Hall JE. Studies on virgulate xiphidiocercariae from Indiana and Michigan. Am Midl Nat. 1960;63: 226–245.

[31] Lang BZ. Note on ecology of *Goniobasis proxima* in North Carolina. Nautilus. 1968;82: 3–5.

[32] Barger MA, Esch GW. *Plagioporus sinitsini* (Digenea: Opecoelidae): a one-host life cycle. J Parasitol. 2000;86: 150–153. 10.1645/0022-3395(2000)086[0150:PSDOAO]2.0.CO;2 10701579

[33] Cable RM. Studies on larval trematodes from Kentucky with a summary of known related species. Am Midl Nat. 1938;19: 440–464.

[34] Horsfall MW. Studies on the structure of Cercaria infracaudata n. sp. J Parasitol. 1930;17: 43–48.

[35] Olson PD, Cribb TH, Tkach VV, Bray RA, Littlewood DTJ. Phylogeny and classification of the Digenea (Platyhelminthes: Trematoda). Int J Parasitol. 2003;33: 733–755. 10.1016/s0020-7519(03)00049-3 12814653

[36] Borcard D, Legendre P. All-scale spatial analysis of ecological data by means of principal coordinates of neighbour matrices. Ecol Modell. 2002;153: 51–68.

[37] Dray S, Legendre P, Peres-Neto PR. Spatial modelling: a comprehensive framework for principal coordinate analysis of neighbour matrices (PCNM). Ecol Modell. 2006;196: 483–493. 10.1016/j.ecolmodel.2006.02.015

[38] Borcard D, Legendre P, Drapeau P. Partialling out the spatial component of ecological variation. Ecology. 1992;73: 1045–1055.

[39] Blanchet FG, Legendre P, Borcard D. Forward selection of spatial explanatory variables. Ecology. 2008;89: 2623–2632. 10.1890/07-0986.1 18831183

[40] Roberts DW. labdsv: Ordination and Multivariate Analysis for Ecology. R package version 1.8–0.; 2016. https://cran.r-project.org/package=labdsv

[41] Oksanen J, Blanchet FG, Kindt R, Legendre P, Minchin PR, O’Hara RB, et al. vegan: Community Ecology Package. R package version 2.3–4; 2016. https://cran.r-project.org/package=vegan

[42] R Core Team. R: A language and environment for statistical computing. Vienna, Austria: R Foundation for Statistical Computing; 2016 https://www.r-project.org/

[43] Sousa WP. Host life history and the effect of parasitic castration on growth: a field study of *Cerithidea californica* Haldeman (Gastropoda: Prosobranchia) and its trematode parasites. J Exp Mar Bio Ecol. 1983;73: 273–296.

[44] Chapuis E. Correlation between parasite prevalence and adult size in a trematode-mollusc system: evidence for evolutionary gigantism in the freshwater snail *Galba truncatula*? J Molluscan Stud. 2009;75: 391–396. 10.1093/mollus/eyp035

[45] Mazerolle MJ. AICcmodavg: Model Selection and Multimodel Inference Based on (Q)AIC(c). R package version 2.0–4; 2016. https://cran.r-project.org/package=AICcmodavg

[46] Arneberg P. An ecological law and its macroecological consequences as revealed by studies of relationships between host densities and parasite prevalence. Ecography. 2001;24: 352–358. 10.1111/j.1600-0587.2001.tb00208.x

[47] Voutilainen A, Van Ooik T, Puurtinen M, Kortet R, Taskinen J. Relationship between prevalence of trematode parasite *Diplostomum* sp. and population density of its snail host *Lymnaea stagnalis* in lakes and ponds in Finland. Aquat Ecol. 2009;43: 351–357. 10.1007/s10452-008-9203-x

[48] Puurtinen M, Knott KE, Suonpaa S, van Ooik T, Kaitala V. Genetic variability and drift load in populations of an aquatic snail. Evolution. 2004;58: 749–756. 10.1111/j.0014-3820.2004.tb00408.x 15154551

[49] Lagrue C, Poulin R. Lack of seasonal variation in the life-history strategies of the trematode, *Coitocaecum parvum*: no apparent environmental effect. Parasitology. 2008;135: 1243–1251. 10.1017/S0031182008004782 18664308

[50] Blasco-Costa I, Waters JM, Poulin R. Swimming against the current: genetic structure, host mobility and the drift paradox in trematode parasites. Mol Ecol. 2012;21: 207–217. 10.1111/j.1365-294X.2011.05374.x 22118193

[51] Heino J, Mykrä H. Control of stream insect assemblages: roles of spatial configuration and local environmental factors. Ecol Entomol. 2008;33: 614–622. 10.1111/j.1365-2311.2008.01012.x

[52] Smith MA, Woodley NE, Janzen DH, Hallwachs W, Hebert PD. DNA barcodes reveal cryptic host-specificity within the presumed polyphagous members of a genus of parasitoid flies (Diptera: Tachinidae). Proc Natl Acad Sci. 2006;103: 3657–3662. 10.1073/pnas.0511318103 16505365 PMC1383497

[53] Thompson RCA. Biology and systematics of *Echinococcus*. Adv Parasitol. 2017;95: 65–109. 10.1016/bs.apar.2016.07.001 28131366

[54] Detwiler JT, Bos DH, Minchella DJ. Revealing the secret lives of cryptic species: Examining the phylogenetic relationships of echinostome parasites in North America. Mol Phylogenet Evol. 2010;55: 611–20. 10.1016/j.ympev.2010.01.004 20064622

[55] Donald KM, Kennedy M, Poulin R, Spencer HG. Host specificity and molecular phylogeny of larval Digenea isolated from New Zealand and Australian topshells (Gastropoda: Trochidae). Int J Parasitol. 2004;34: 557–568. 10.1016/j.ijpara.2003.11.027 15064120

[56] Leung TLF, Keeney DB, Poulin R. Cryptic species complexes in manipulative echinostomatid trematodes: when two become six. Parasitology. 2009;136: 241–52. 10.1017/S0031182008005374 19091157

[57] Locke SA, Daniel McLaughlin J, Marcogliese DJ. DNA barcodes show cryptic diversity and a potential physiological basis for host specificity among Diplostomoidea (Platyhelminthes: Digenea) parasitizing freshwater fishes in the St. Lawrence River, Canada. Mol Ecol. 2010;19: 2813–2827. 10.1111/j.1365-294X.2010.04713.x 20561195

[58] Miura O, Kuris AM, Torchin ME, Hechinger RF, Dunham EJ, Chiba S. Molecular-genetic analyses reveal cryptic species of trematodes in the intertidal gastropod, *Batillaria cumingi* (Crosse). Int J Parasitol. 2005;35: 793–801. 10.1016/j.ijpara.2005.02.014 15925598

[59] Barker SC, Blair D, Garrett AR, Cribb TH. Utility of the D1 domain of nuclear 28S rRNA for phylogenetic inference in the Digenea. Syst Parasitol. 1993;26: 181–188. 10.1007/BF00009725

[60] Herrmann KK, Poulin R, Keeney DB, Blasco-Costa I. Genetic structure in a progenetic trematode: Signs of cryptic species with contrasting reproductive strategies. Int J Parasitol. 2014;44: 811–818. 10.1016/j.ijpara.2014.06.006 25058509

[61] Miller TL, Cribb TH. Two new cryptogonimid genera (Digenea, Cryptogonimidae) from *Lutjanus bohar* (Perciformes, Lutjanidae): analyses of ribosomal DNA reveals wide geographic distribution and presence of cryptic species. Acta Parasitol. 2007;52: 104–113. 10.2478/s11686-007-0019-y

[62] Blasco-Costa I, Balbuena JA, Raga JA, Kostadinova A, Olson PD. Molecules and morphology reveal cryptic variation among digeneans infecting sympatric mullets in the Mediterranean. Parasitology. 2010;137: 287–302. 10.1017/S0031182009991375 19849887

[63] Nadler SA, De León GP-P. Integrating molecular and morphological approaches for characterizing parasite cryptic species: implications for parasitology. Parasitology. 2011;138: 1688–1709. 10.1017/S003118201000168X 21281559

[64] Blasco-Costa I, Poulin R. Host traits explain the genetic structure of parasites: a meta-analysis. Parasitology. 2013;140: 1316–1322. 10.1017/S0031182013000784 23866918

[65] Criscione CD, Blouin MS. Life cycles shape parasite evolution: comparative population genetics of salmon trematodes. Evolution. 2004;58: 198–202. 10.1111/j.0014-3820.2004.tb01587.x 15058733

[66] Louhi K-R, Karvonen A, Rellstab C, Jokela J. Is the population genetic structure of complex life cycle parasites determined by the geographic range of the most motile host? Infect Genet Evol. 2010;10: 1271–1277. 10.1016/j.meegid.2010.08.013 20804859

[67] Prugnolle F, Théron A, Pointier JP, Jabbour-Zahab R, Prugnolle F, Thron A, et al Dispersal in a parasitic worm and its two hosts: consequence for local adaptation. Evolution. 2005;59: 296–303. 15807416

