# Supplementary material for: Spatial scale and structure of complex life cycle trematode parasite communities in streams
Source: PLoS One. 2020 Nov 24;15(11):e0241973. doi: 10.1371/journal.pone.0241973 (PMC7685432; doi:10.1371/journal.pone.0241973)
Supplement: S1 Appendix — (DOCX) [file pone.0241973.s001.docx]

**S1 Appendix**

**Materials and methods**

**Study sites**

*Landscape-level study*: Distance between the 20 sites ranged from < 1 km to 132 km. All sites were first, second or third order streams in the Upper New River Basin and ranged in mean width from 1.5 to 9.85 m. Surrounding land use, canopy cover and substrate composition varied across sites from completely closed reaches in forests with predominately cobble substrate, to mostly open reaches in agricultural grazing land with predominately silt and sand substrate. Site elevation ranged from 673 to 952 m. Sites were chosen to comprise a range of *E. proxima* densities (based on Dillon 1982 and preliminary samples collected in 2009).

*Within-network study*: The two main stem (third order) sites were open canopy with predominately cobble substrate and ranged in mean width from 9.8 to 12.4 m. Main stem sites ranged in elevation from 684 to 705 m and were separated by 5.5 km in-stream distance (3.4 km Euclidean distance). The six headwater sites (first or second order streams) were closed canopy with predominately cobble substrate and ranged in mean width from 1.3 to 3.2 m. Headwater sites ranged in elevation from 726 to 876 m, and were separated from the first main stem site (farthest downstream) by a minimum of 2.6 km in-stream distance (2.1 km Euclidean distance) and a maximum of 13 km in-stream distance (6.1 km Euclidean distance).

**Molecular identification of trematodes**

Prior to DNA extraction, we soaked tissue twice in Milli-Q water to remove ethanol, and transferred samples in 2 µl of Milli-Q water into 0.2 ml tubes. For DNA extraction, we added 98 µl of 6.7% Chelex and 1% Proteinase K solution and incubated tubes at 56°C for 2 hours, followed by an 8 min boil at 100°C. We amplified DNA with PCR using forward primer LSU-5 and reverse primer 1500R (Olson et al. 2003). Each 25 µl reaction comprised 3 µl of DNA sample, 0.5 µl each of 20µM forward and reverse primers, and 12.5 µl of PCR master mix (GoTaq G2 Hot Start Colorless Master Mix, Promega Corp., Madison, WI). Thermal cycling parameters for PCR included an initial denaturation (3 minutes at 95°C), 40 cycles of amplification (45 seconds at 94°C, 30 seconds at 54°C and 2 minutes at 72°C), and a final extension period (7 minutes at 72°C). Amplicons were visualized using gel electrophoresis. PCR products were cleaned with a QIAquick PCR Purification kit (Qiagen Inc., Valencia, CA) following the manufacturer’s instructions and eluted with 30 µl Milli-Q water. Purified samples were sequenced with the same PCR primers. We aligned sequences in Geneious v. 8.0.4 (Biomatters Ltd., Auckland, New Zealand).

**Results**

**Trematode prevalence and diversity**

*Landscape-level study*: *Metagonimoides oregonensis* ranged in prevalence from 2% (Long Branch) to 20% (Francis Mill), while virgulate infections were less prevalent and ranged from < 1% (Chisholm and West Fork Dodd Creeks) to 10% (East Fork Crooked Creek). Cotylomicrocercous type infections were also common (encountered at 17 out of 20 sites) and comprised the highest prevalence of any single type of infection with 38% of snails at one site (Little Wilson) infected. Infections with other trematode types were rare; *Sanguinicola* sp. was present at three sites with a maximum prevalence of 6% (Furnace Creek), and monostome type cercariae were present at one site with a prevalence of 4% (Cherry Creek).

All unique haplotypes were BLAST searched in GenBank on May 5, 2016. For cotylomicrocercous type infections, we identified 9 unique haplotypes, all of which most closely matched (max identity 97 – 99%) *Plagiocirrus loboides* (Opecoelidae). Among these sequences, haplotype divergence within a 1,265 bp fragment ranged from 1 to 51 bp (0.08 – 4.0%). For infections visually identified as *M. oregonensis*, we recovered seven haplotypes. Six of these haplotypes most closely matched (max identity 98 – 99%) *Metagonimoides oregonensis* (Heterophyidae) and varied at 1 to 17 sites within a 1,293 bp fragment (0.08 – 1.31% divergence). The seventh haplotype was most similar to (max identity 97%) *Clonorchis sinensis* (Opisthorchiidae), and differed from *M. oregonensis* haplotypes by 83 to 88 bp (6.4 – 6.75% divergence).

From virgulate infections, we identified eight haplotypes most similar to four species from three different families. Four of these haplotypes most closely matched species within the family Lecithodendriidae: three haplotypes most closely matched (max identity 97%) *Paralecithodendrium parvouterus* and one most closely matched (max identity 97%) *Lecithodendrium linstowi*. Divergence among these haplotypes within a 1,242 bp fragment ranged from 6 to 65 base pairs (0.48 – 5.23%). Two other virgulate haplotypes most closely matched (max identity 96%) *Collyriclum faba* (Collyriclidae), and were variable at 1 site within a 1,287 bp fragment (0.08% divergence). The remaining two virgulate haplotypes most closely matched (max identity 92 – 93%) *Allassogonoporus amphoraeformis* (Pleurogenidae), and were variable at 4 sites within a 1,282 bp fragment (0.31% divergence).

We identified a single haplotype each for *Sanguinicola* sp. and monostome type infections: respectively, these were most similar to (max identity 85%) *Sanguinicola cf. inermis* (Aporocotylidae) and (max identity 98%) *Notocotylus* sp. (Notocotylidae). Additionally, we found four unique haplotypes from infections with immature or no cercariae, which included: one additional haplotype that most closely matched (max identity 97%) *Plagiocirrus loboides* (Opecoelidae); one haplotype that was most similar to (max identity 99%) multiple sequences within Echinostomatidae; and two haplotypes that most closely matched (max identity 94%) additional species within Pleurogenidae, *Collyricloides massanae* and *Parabascus duboisi*.

*Within-network study*: Small virgulate infections were present at 6 of 8 sites and comprised the highest prevalence of infection at a single site at 42.1% (AB3). Large virgulate infections were encountered at 6/8 sites with a maximum prevalence of 5.8% (BB2). Cotylomicrocercous type infections were present at 5/8 sites with a maximum prevalence of 15.3% (BB1).

For *M. oregonensis* infections, we identified two haplotypes, both of which most closely matched (max identity 98 – 99%) *Metagonimoides oregonensis* (Heterophyidae). The two *M. oregonensis* haplotypes differed at 14 sites across a 1,293 bp fragment (1.08% divergence). From small virgulate infections, we identified two haplotypes, both of which most closely matched (max identity 96 – 97%) *Paralecithodendrium parvouterus* (Lecithodendriidae). The two small virgulate haplotypes varied at 19 sites across a 1,142 bp fragment (1.66% divergence). We identified two additional haplotypes from large virgulate infections that most closely matched two different species, (max identity 94%) *Collyricloides massanae* and (max identity 92%) *Allassogonoporus amphoraeformis*, both within the family Pleurogenidae. For cotylomicrocercous type infections, we identified a single haplotype matched (max identity 99%) to *Plagiocirrus loboides* (Opecoelidae).

**References**

Dillon Jr., R. T. 1982. The correlates of divergence in isolated populations of the freshwater snail, *Goniobasis proxima*. Ph.D. Dissertation, University of Pennsylvania, Philadelphia, Pennsylvania.

Olson, P. D. et al. 2003. Phylogeny and classification of the Digenea (Platyhelminthes: Trematoda). – Int. J. Parasitol. 33: 733–755.

**S1 Table.** Physicochemical, snail density and trematode infection data for the 20 streams within the Upper New River Basin in the landscape-level study. Stream width (W), specific conductance (TCC), total phosphorus (TP), total nitrogen (TN) and elevation (Elev). Trematode morphotypes: cotylomicrocercous (COTYL); *M. oregonensis* (META); virgulate (VIRG); *Sanguinicola* sp. (SANG); and monostome type (MONO).

**S2 Table.** Geographic coordinates for the 20 streams within the Upper New River Basin in the landscape-level study.

**S3 Table.** Physicochemical, snail density and trematode infection data for 8 study sites in Big Reed Island Creek drainage in Carroll Co., VA, in the within-network study. Stream width (W), maximum depth (D), discharge (Q), specific conductance (TCC), dissolved oxygen (DO). *Metagonimoides oregonensis* (META), small virgulate morphotype (SVIRG), large virgulate morphotype (LVIRG), and cotylomicrocercous morphotype (COTYL).

**S4 Table.** Geographic coordinates for the 8 study sites in Big Reed Island Creek drainage in Carroll Co., VA, in the within-network study.

**S5 Table.** Variation partitioning of community composition for morphotype- and haplotype-defined communities in the landscape-level study. Adjusted R^2^ is the fraction of variation in community composition explained by environmental (E) or spatial (S) variables. Pure E is the variation explained by environmental variables alone, S str. E is the variation explained by spatially structured environmental variables, Pure S is the variation explained by spatial variables alone, and E + S is the total variation explained by environmental and spatial variables combined.

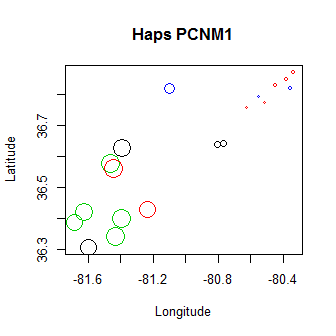

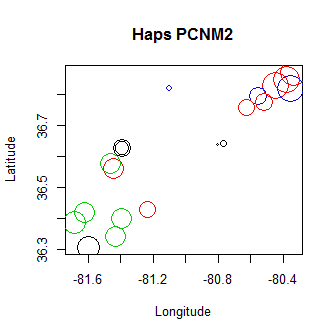


1. PCNM2
2. PCNM1

**(c) PCNM3**


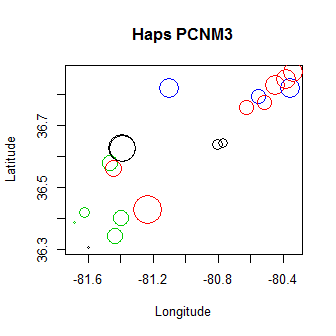


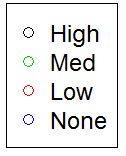


**S1 Fig**. Maps representing significant spatial variables (eigenvectors calculated using principal coordinate analysis of neighbor matrices, PCNM) used in variation partitioning of trematode communities in the landscape-level study. Point size is proportional to the absolute value of the spatial filter at the site. Color corresponds to prevalence of cotylomicrocercous type infections at that site, categorized as: High > 10%; Medium = 5 – 10%; and Low < 5% of snails infected. Note that the scale of the spatial gradient described by a PCNM filter decreases with increasing PCNM number.
